# Detection and Prevention of Mild Cognitive Impairment and Dementia

**DOI:** 10.3390/healthcare11162232

**Published:** 2023-08-08

**Authors:** Lambros Messinis, Grigorios Nasios, Panagiotis Ioannidis, Panayiotis Patrikelis

**Affiliations:** 1 Laboratory of Cognitive Neuroscience, School of Psychology, Aristotle University of Thessaloniki, 54124 Thessaloniki, Greece; 2 Department of Speech and Language Therapy, School of Health Sciences, University of Ioannina, 45500 Ioannina, Greece; grigoriosnasios@gmail.com; 3 B’ Department of Neurology, AHEPA University Hospital, 1st Kyriakides Str., Aristotle University, 54124 Thessaloniki, Greece

Mild cognitive impairment (MCI) is characterized by cognitive deficits alongside essentially preserved competence in activities of daily living. It is a risk factor for the development of dementia and can reflect a prodromal predementia state of Alzheimer’s disease (AD), vascular dementia (VD), Parkinson’s disease dementia (PDD), and other degenerative dementias [1]. Early symptoms of MCI and dementia may not be apparent in routine clinical examinations and are sometimes even concealed during routine clinical visits for other complaints. Although current methods of detecting moderate dementia in community-based clinical practices are reasonably accurate, they do not sensitively detect MCI and often do not detect mild dementia. The difficulty of detecting MCI and, in many cases, mild dementia is largely due to the insensitivity of the most commonly used screening tests in clinical practice, e.g., the Mini Mental Status Examination (MMSE) [2]. This insensitivity is caused by a person with MCI or very mild dementia experiencing subtle memory problems only slightly greater than those normally expected with aging without showing any other symptoms of dementia, such as impaired judgment or reasoning. The insensitivity is even more significant in illiterate individuals, whose poor memory performance appears to be attributed both to the nature of the task and the use of different cognitive mechanisms to recall learned information [3].

Given that early detection is critical for treatment, effective methods of screening for MCI and dementia are high priorities in research. Therefore, more sensitive screening tests or small and practical neuropsychological batteries are needed in community healthcare settings. Moreover, due to advances in the field of biomarker-based early detection of MCI and dementia, it is now possible to differentiate between MCI patients with and without underlying pathological conditions, and thus between patients with a low and high risk of developing dementia [4]. However, the current guidelines and recommendations in many countries (in Europe and worldwide) for the diagnostic use of biomarkers in predementia detection are limited and somewhat unclear [5].

As the population ages, there is a growing need for early, proactive programs that can delay the consequences of dementia and improve the quality of life of people with MCI and their caregivers. Both pharmacological and non-pharmacological approaches (cognitive stimulation/rehabilitation, nutritional supplementation, physical exercise, electric/magnetic stimulation), as well as multicomponent interventions, have been proposed [6]. Various nonpharmacological interventions for older people with MCI have been conducted and are found to delay cognitive deterioration [7,8]. Similar nonpharmacological interventions have also been applied to patients with early-stage Alzheimer’s disease, with beneficial effects on several cognitive domains and everyday functioning capacity [9,10].

However, several issues have not been adequately addressed in the international literature, including which screening instruments or brief neuropsychological batteries can best detect dementia or mild cognitive impairment (MCI) in community-dwelling older patients undergoing primary care. Secondly, does the combined use of screening tests, brief neuropsychological batteries, and diagnostic biomarkers improve the accuracy of detecting MCI and mild dementia in outpatient and other clinical settings compared with using tests alone? Finally, do early nonpharmacological or combined interventions for cognitive impairment in community-dwelling older adults or patients diagnosed with MCI improve decision making and patient/family/caregiver outcomes?

In this Special Issue of *Healthcare*, we present valuable findings in the form of three innovative articles in the field. In the first study, Roeul Kim and Woojin Chung [11] examined the effect of aging on gender-specific educational differences in the risk of cognitive impairment using a nationally representative sample of 4278 men and 5495 women aged 45 years and older from the dataset of the Korean Longitudinal Study of Aging. They found that the prevalence of cognitive impairment was higher in women than in men at baseline and that the risk of cognitive impairment in each age group decreased with education level for both genders. Moreover, the risk of cognitive impairment was worse for those with a lower level of education and increased with age, more so for women than for men. The authors conclude that aging appears to widen the impact of educational differences on the risk of cognitive impairment and is more unfavorable for women than for men, at least in Korea. The authors further highlight that, regarding population aging in Korea, public health policy makers need to take note of their findings and make efforts to identify the target population to reduce both the level and difference in the risk of cognitive impairment.

In the second article, Messinis et al. provide [12] data on a new and sensitive cognitive screening instrument (CSI), which is essential for everyday practice. This instrument, known as “The Quick Mild Cognitive Impairment (Qmci) screen”, is designed to identify mild cognitive impairment, and its content was recently translated into Greek (Qmci-Gr). In their study, Messinis et al. compared the diagnostic values of cognitive impairment against the Montreal Cognitive Assessment (MoCA) screen and examined its optimal cutoffs. The researchers recruited consecutive patients aged ≥55 years that presented with cognitive complaints from two outpatient clinics in Greece. A total of 145 patients, with a median age of 70 years, were assessed; 44 were classified as having subjective memory complaints (SMC) but normal cognition, 32 had MCI, and 69 had dementia. The authors found that the Qmci-Gr had a higher accuracy compared to the MoCA in discriminating MCI from dementia, and its accuracy was marginally higher for distinguishing SMC from dementia. In contrast, they found that the Qmci-Gr presented a lower accuracy than the MoCa in differentiating SMC from MCI. The authors conclude that the Qmci-Gr has a diagnostic accuracy comparable to the MoCA regarding MCI and dementia groups and may be a useful cognitive screening tool in everyday clinical practice.

In the third article, Chalkias et al. [13] provide an sophisticated review that provides insights into optical coherence tomography angiography (OCTA), a non-invasive imaging modality used to visualize retinal layers and vessels, showing encouraging results for the study of various neurological conditions, including Alzheimer’s disease and vascular dementia. The authors argue that, according to the current literature, vessel density seems to be a common biomarker for both aforementioned forms of dementia. They further stipulate that inner retinal layer thickness might represent a biomarker preferentially affected in degenerative dementia, including Alzheimer’s disease, while, in contrast, the outer layer thickness as a whole justifies attention as a potential vascular dementia biomarker. They further recommend that radial peripapillary capillary density should also be studied as a biomarker specifically linked to vascular dementia.

The articles published in this Special Issue represent only a very small aspect of the total research that must be conducted to adequately address and verify the most pressing clinical issues. However, we are confident that these articles provide important new information and further illuminate the need for further research. We close this editorial with the hope that future Special Issues may provide the scientific community with further empirical research and evidence related to the detection and prevention of mild cognitive impairment and dementia.
